# Nasal High Flow in Room Air for Hypoxemic Bronchiolitis Infants

**DOI:** 10.3389/fped.2019.00426

**Published:** 2019-10-25

**Authors:** Donna Franklin, Franz E. Babl, Kristen Gibbons, Trang M. T. Pham, Nadia Hasan, Luregn J. Schlapbach, Ed Oakley, Simon Craig, Jeremy Furyk, Jocelyn Neutze, Susan Moloney, John Gavranich, Prasanna Shirkhedkar, Vishal Kapoor, Simon Grew, John F. Fraser, Stuart Dalziel, Andreas Schibler, PARIS and PREDICT

**Affiliations:** ^1^Paediatric Critical Care Research Group, Queensland Children's Hospital, The University of Queensland, Brisbane, QLD, Australia; ^2^School of Medicine, The University of Queensland, Brisbane, QLD, Australia; ^3^Mater Research Institute, The University of Queensland, Brisbane, QLD, Australia; ^4^Critical Care Research Group, Adult Intensive Care Service, The Prince Charles Hospital, Brisbane, QLD, Australia; ^5^Paediatric Research in Emergency Departments International Collaborative (PREDICT), Parkville, VIC, Australia; ^6^Royal Children's Hospital, Emergency Department, Melbourne, VIC, Australia; ^7^Murdoch Children's Research Institute, Melbourne, VIC, Australia; ^8^Department of Paediatrics, Faculty of Medicine, Dentistry and Health Sciences, University of Melbourne, Melbourne, VIC, Australia; ^9^Department of Medicine, School of Clinical Sciences, Monash University, Clayton, VIC, Australia; ^10^Monash Medical Centre, Emergency Department, Melbourne, VIC, Australia; ^11^College of Medicine and Dentistry, James Cook University, Townsville, QLD, Australia; ^12^The Townsville Hospital, Emergency Department, Townsville, QLD, Australia; ^13^KidzFirst Middlemore Hospital, Auckland, New Zealand; ^14^University of Auckland, Auckland, New Zealand; ^15^Department of Paediatrics, Gold Coast University Hospital, Southport, QLD, Australia; ^16^School of Medicine, Griffith University, Gold Coast, QLD, Australia; ^17^Faculty of Health Sciences and Medicine, Bond University, Gold Coast, QLD, Australia; ^18^Paediatric Department, Ipswich General Hospital, Ipswich, QLD, Australia; ^19^Paediatric Department, Caboolture Hospital, Caboolture, QLD, Australia; ^20^Paediatric Department, Redcliffe Hospital, Redcliffe, QLD, Australia; ^21^Children's Emergency Department, Starship Children's Hospital, Auckland, New Zealand; ^22^Liggins Institute, University of Auckland, Auckland, New Zealand

**Keywords:** oxygen therapy, room air, bronchiolitis, respiratory illness, nasal high flow therapy

## Abstract

**Background:** Bronchiolitis is the most common reason for hospital admission in infants, with one third requiring oxygen therapy due to hypoxemia. It is unknown what proportion of hypoxemic infants with bronchiolitis can be managed with nasal high-flow in room air and their resulting outcomes.

**Objectives and Settings:** To assess the effect of nasal high-flow in room air in a subgroup of infants with bronchiolitis allocated to high-flow therapy in a recent multicenter randomized controlled trial.

**Patients and Interventions:** Infants allocated to the high-flow arm of the trial were initially treated with room air high-flow if saturations were ≥85%. Subsequently, if oxygen saturations did not increase to ≥92%, oxygen was added and FiO_2_ was titrated to increase the oxygen saturations. In this planned sub-study, infants treated during their entire hospital stay with high-flow room air only were compared to infants receiving either standard-oxygen or high-flow with oxygen. Baseline characteristics, hospital length of stay and length of oxygen therapy were compared.

**Findings:** In the per protocol analysis 64 (10%) of 630 infants commenced on high-flow room air remained in room air only during the entire stay in hospital. These infants on high-flow room air were on average older and presented with moderate hypoxemia at presentation to hospital. Their length of respiratory support and length of stay was also significantly shorter. No pre-enrolment factors could be identified in a multivariable analysis.

**Conclusions:** In a small sub-group of hypoxemic infants with bronchiolitis hypoxemia can be reversed with the application of high-flow in room air only.

**Trial registration:** ACTRN12615001305516

## Introduction

The application of oxygen for hypoxemia represents one of the most common medical and nursing interventions globally (1). Traditionally, oxygen was considered a safe intervention, but an increasing body of evidence demonstrates potential for harm in acute care settings including perinatal adaptation (2, 3), cardiac arrest (4), and acute stroke (5). Adverse effects of oxygen may relate to toxicity of free oxygen radicals, and adaptive mechanisms in health and disease (6–8). As a result, the application of oxygen as a drug is undergoing more scrutiny. Furthermore, access to oxygen in remote facilities and in low income countries can be problematic (9, 10).

The oxygen requirement in acute respiratory diseases leading to hypoxemia depends on several pathophysiological factors such as ventilation inhomogeneity, atelectasis, or consolidated lung parenchyma, an increased intra pulmonary shunt fraction and decreased alveolar-capillary membrane diffusion capacity (11). Traditionally, the first approach to improve hypoxemia consisted in increasing inspired oxygen fraction to treat the symptom whilst simultaneously searching for the cause of the hypoxemia. If this approach is ineffective then non-invasive, mainly continuous positive airway pressure (CPAP) or invasive ventilation strategies are commonly used in an incremental fashion, though both are highly resource and skill dependent (12). In contrast to oxygen alone, alveolar recruitment by provision of CPAP is likely to reverse or improve many conditions that lead to hypoxemia in children, whereas restricted oxygen diffusion due to thickened alveolar-capillary membrane is less commonly a feature early in the disease process. The application of CPAP however can be challenging, requires highly trained nursing and medical staff, is not always tolerated by patients, and therefore has been in the past restricted to intensive care unit (ICU) settings.

Nasal high-flow therapy, as a new positive airway pressure modality has been rapidly adopted in ICU, and subsequently in emergency departments (EDs) and general wards because of its ease of use (13–15). Physiological and clinical studies demonstrate a CPAP effect at high-flow rates that exceed peak inspiratory flow (16–18).

We recently published the findings of the Pediatric Acute Respiratory Intervention (PARIS) randomized controlled trial (RCT), which randomized 1,472 hypoxemic infants with bronchiolitis to either nasal high-flow therapy or standard-oxygen therapy with the option to use rescue high-flow if infants failed standard-oxygen therapy (19). Nested within this RCT we addressed the pre-planned question whether high-flow therapy can normalize oxygenation without any increase in the F_i_O_2_ provided (20). We hypothesized that high-flow room air is sufficient to restore adequate oxygenation in a subset of infants with bronchiolitis presenting with hypoxemia.

## Methods

The protocol, methods, and results of the RCT have been described previously (19, 20). Briefly, 1,472 infants, <12 months of age, admitted to 17 hospitals in Australia and New Zealand with bronchiolitis and an oxygen requirement were randomized in an open-label study design to treatment with standard subnasal oxygen or nasal high-flow (20). The primary outcome for the trial was treatment failure, defined by the presence of ≥3 of a possible 4 abnormal physiological parameters, and escalation of care. The trial confirmed that treatment failure and escalation of care occurred in fewer patients randomized to high-flow compared with standard-oxygen (*p* < 0.001). The trial was undertaken between October 2013 and August 2016, and the relevant human research ethics committee at each participating site approved the study (HREC/13/QRCH/93). Informed written consent was obtained by parent or guardian.

The primary objective of this pre-specified sub-study was to test if the oxygen requirement can be reversed in infants with bronchiolitis, by the provision of room air high-flow alone. For this purpose, the study protocol required that all infants, who were allocated to the high-flow arm, and with enrolment oxygen saturation between 85 and≤92/94%, to be initially commenced on high-flow room air (FiO_2_ 0.21) at 2 L/kg/min via age-appropriate Optiflow Junior^TM^ cannula and Airvo2^TM^ high-flow system (Fisher & Paykel Healthcare; Auckland, New Zealand). If high-flow room air had failed to achieve target saturations of ≥92% in participating tertiary children's hospitals, and ≥94% in secondary hospitals (saturation targets were set by individual institution's standard current practice and not specified centrally by the trial investigators) after 10 min the F_i_O_2_ of the high-flow system was increased to achieve the target saturation. High-flow room air was stopped (i.e., flow rate reduced from 2 to 0 L/kg/min without gradual reduction) as soon as clinicians saw clinical improvement and participant's oxygen saturation was maintained at ≥92/94% for at least 4 h. For those infants receiving nasal high-flow oxygen, the F_i_O_2_ was titrated to keep saturations between a 92/94 and 98% target, and once on high-flow room air for 4 h and maintaining target saturations, high-flow therapy was stopped. If infants subsequently developed a further oxygen requirement, according to their institution's definition, high-flow was instigated using the same procedures as at initiation of high-flow at randomization. Infants allocated to standard-oxygen therapy received 100% subnasal oxygen up to a maximum of 2 L/min to maintain oxygen saturations within the same targets as the high-flow arm. All other bronchiolitis specific therapies were at the discretion of the treating clinician.

### Oxygen Therapy Groups (Figure 1)

We defined four oxygen therapy groups for this sub-study. Infants allocated to standard-oxygen remained on subnasal standard-oxygen (standard-oxygen group) unless they required escalation of care, which in most of these infants was achieved by using high-flow with oxygen. Infants allocated to high-flow with saturations <85% at randomization received immediate high-flow with oxygen (high-flow with immediate oxygen group). The threshold of 85% was chosen in consensus with the participating hospitals. Infants allocated to high-flow presenting with saturations between 85 and≤92/94% were started on high-flow room air. Infants without improved oxygenation (saturation≤92/94%) after 10 min on high-flow room air were switched to high-flow with oxygen (high-flow with oxygen group) whereas infants who improved their saturations to ≥92/94% remained on high-flow room air (high-flow room air group).

**Figure 1:**
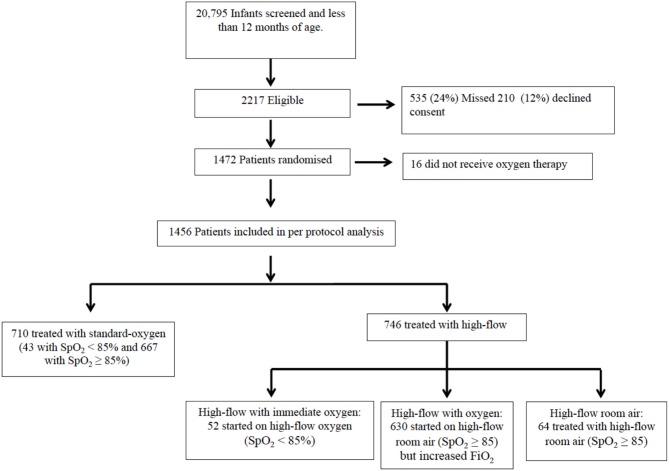
Oxygen treatment group allocation.

### Study Outcomes

The primary outcomes were: first a proof of concept to demonstrate whether a subgroup of hypoxemic infants with bronchiolitis can be treated with high-flow room air only while maintaining their target saturations; and secondly to determine the proportion of hypoxemic infants with bronchiolitis that respond to high-flow in room air. Secondary outcomes included differences in basic demographics, risk factors such as prematurity and pre-existing disease, virus detected, severity of disease at randomization, length of hospital stay, and length of high-flow therapy. The physiological response of heart and respiratory rate to high-flow room air was analyzed for the first 3 days of admission. In one of the participating hospitals all data on the change of respiratory and heart rate, and saturation of all infants enrolled were also obtained relating to the first hour of oxygen therapy post start of the intervention (**Table 2**).

The sub-group of infants receiving high-flow room air only were compared to the three oxygen groups; infants on standard oxygen, infants on high-flow with immediate oxygen, and infants high-flow with oxygen.

### Statistical Analysis

The sample size of 1,400 infants for the RCT was based on a 50% reduction in failure rate of oxygen therapy at 90% power and type I error of 0.05. Allocation to treatment arm was as the per protocol analysis. Continuous data are presented as mean and standard deviation (SD) or median and interquartile range (IQR) dependent on variable distribution; categorical data are presented using number and percentage. Comparisons between the treatment groups were made using ANOVA, Kruskal–Wallis or Fisher's exact tests; following a significant *p*-value (<0.05) *post-hoc* comparisons were undertaken comparing the in-room air group to the other treatment groups (adjustment using Bonferroni's correction). Further analysis was undertaken comparing high-flow in room air vs. a combined group of standard-oxygen and high-flow with oxygen. Bivariable analysis was initially undertaken and variables with a *p* <0.25 remained in the multivariable model. Comparison of infants in the standard oxygen group presenting with pre-enrolment saturations <85% to infants with saturations between 85 and≤92/94% were undertaken using *t*-tests, Mann–Whitney *U*-test and Fisher's exact test. Analyses were conducted in StataIC version 14.1 (StataCorp LP, College Station, Texas).

## Results

**Figure 2:**
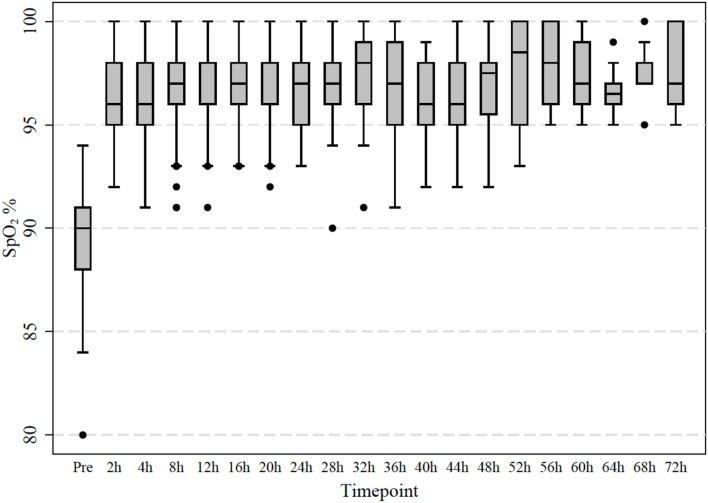
Measured transcutaneous saturation of oxygen levels in 64 infants treated with room air high-flow. Observations were taken initially after 2 h and then every 4 h.

**Figure 3:**
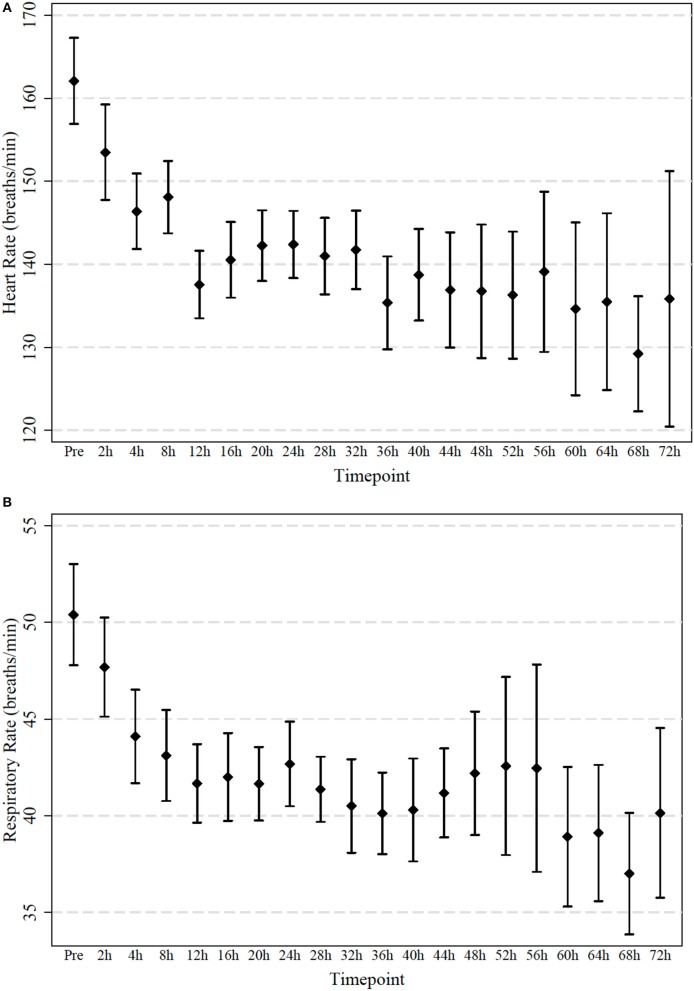
**(A,B)** Measured heart and respiratory rate in 64 infants treated with high-flow in room air. Observations were taken initially after 2 h and then every 4 h. Mean and 95% confidence interval are indicated.

In the original RCT, 1,472 infants with bronchiolitis were randomized and subsequently provided written consent; in the per protocol analysis 710 received standard-oxygen and 746 received high-flow of which 52 (7%) commenced on high-flow with immediate oxygen, 630 (70%) commenced high-flow room air with a later increase of FiO_2_ and 64 (9%) received high-flow room air (Figure 1). Table 1 shows the baseline characteristics of the infants in high-flow room air compared to infants on high-flow with immediate oxygen, high-flow with oxygen and infants treated with standard-oxygen therapy. There were a number of statistical differences in several of the pre-enrolment factors between the three study groups (Table 1): the distribution of age at enrolment was significantly different between the treatment groups with the proportion of infants <3 months of age lowest in the high-flow room air (17.2%), the body weight was highest in the high-flow room air group, followed by the standard-oxygen group and then high-flow in oxygen. Infants on high-flow with immediate oxygen received more frequently antibiotics and sedation. Infants in the high-flow room air group showed higher oxygen saturation pre-enrolment than infants in the other groups (Table 2). In the cohort of infants (*n*=334) in which physiological parameters were measured within the first hour of treatment, infants on standard-oxygen showed the highest saturations. The changes in saturation, heart and respiratory rate in infants on high-flow room air are shown for the first 72 h in Figures 2, 3.

**Table 1:** Pre-enrolment characteristics.

**Pre-enrolment characteristic**		**Treatment group**				***p*-value**
		**Standard-oxygen**	**High-flow with immediate oxygen**	**High-flow with oxygen**	**High-flow room air**	
		***N*** **=** **710**	***N*** **=** **52**	***N*** **=** **630**	***N*** **=** **64**	
Age (months)^∧^		6.3 (6.1)	3.5 (6.6)	5.8 (6.3)	5.9 (4.5)	0.001
Age (months)	≤3 months	180 (25.4)	24 (46.2)	180 (28.6)	11 (17.2)	0.002
	>3–6 months	160 (22.5)	13 (25.0)	154 (24.4)	22 (34.4)	
	>6 months	370 (52.1)	15 (28.9)	296 (47.0)	31 (48.4)	
Weight (kg)^#^		7.62 (2.22)	6.45 (2.39)	7.27 (2.24)	7.46 (1.96)^*^	<0.001
Sex	Female	252 (35.5)	17 (32.7)	251 (39.8)	22 (34.4)	0.339
Ethnicity	Caucasian	370 (52.1)	20 (38.5)	343 (54.4)	29 (45.3)	0.213
	Aboriginal/Torres Strait Islander	29 (4.1)	2 (3.9)	24 (3.8)	3 (4.7)	
	Maori/Pacific Islander	213 (30.0)	18 (34.6)	157 (24.9)	22 (34.4)	
	Other/unknown	98 (13.8)	12 (23.1)	157 (24.9)	10 (15.6)	
Risk factors	Prematurity <37 weeks	123 (17.3)	11 (21.2)	118 (18.6)	11 (17.2)	0.840
	Previous need for neonatal respiratory support	101 (14.2)	11 (21.2)	93 (14.8)	11 (17.2)	0.506
	Oxygen only	36 (5.1)	3 (5.8)	24 (3.8)	3 (4.7)	0.608
	CPAP/NIV	70 (9.9)	8 (15.4)	60 (9.5)	8 (12.5)	0.454
	Invasive ventilation	19 (2.7)	3 (5.8)	21 (3.3)	5 (7.8)	0.087
	Other respiratory support	12 (1.7)	3 (5.8)	19 (3.0)	3 (4.7)	0.059
	Previous hospital admissions for respiratory disease postnatal	217 (30.6)	14 (26.9)	160 (25.4)	14 (21.9)	0.140
	Previous ICU admission for respiratory support	46 (6.5)	2 (3.9)	21 (3.3)	3 (4.7)	0.057
	Intubated & ventilated	7 (1.0)	0 (0.0)	4 (0.6)	0 (0.0)	0.822
	Mask ventilation	6 (0.9)	0 (0.0)	2 (0.3)	0 (0.0)	0.638
	High-flow therapy	35 (4.9)	2 (3.9)	15 (2.4)	2 (3.1)	0.081
	Chronic Lung Disease	12 (1.7)	0 (0.0)	14 (2.2)	3 (4.7)	0.273
	Congenital Heart Disease	16 (2.3)	0 (0.0)	8 (1.3)	0 (0.0)	0.391
	Patient history of wheeze	174 (24.7)	9 (17.3)	133 (21.3)	15 (23.8)	0.377
	Family history of asthma	349 (49.9)	17 (32.7)	286 (46.4)	31 (50.8)	0.082
	Family history of allergy	159 (22.8)	8 (15.4)	111 (18.0)	14 (23.0)	0.130
	Currently attending child care?	91 (13.2)	6 (11.5)	77 (12.8)	11 (18.0)	0.671
Viral etiology	Number tested	*N*=564	*N*=41	*N*=536	*N*=45	–
	Respiratory syncytial virus	309 (54.8)	30 (73.2)	287 (53.5)	25 (55.6)	0.110
	Other viruses	194 (34.4)	11 (26.8)	155 (28.9)	14 (31.1)	0.241
	Multiple viruses	106 (18.8)	10 (24.4)	86 (16.0)	9 (20.0)	0.372
	No virus detected on nasopharyngeal aspirate	108 (19.2)	5 (12.2)	134 (25.0)	11 (24.4)	0.047
Non study treatments received	Any non-study treatments received	510 (71.8)	46 (88.5)^*^	410 (65.1)	44 (68.8)	0.001
	Steroids	59 (8.3)	1 (1.9)	50 (7.9)	1 (1.6)	0.089
	Nebulized Saline	87 (12.3)	7 (13.5)	70 (11.1)	3 (4.7)	0.281
	Bronchodilators	211 (29.7)	12 (23.1)	158 (25.1)	10 (15.6)	0.036
	Adrenaline nebulizations	2 (0.3)	1 (1.9)	5 (0.8)	0 (0.0)	0.211
	Antibiotics	115 (16.2)^*^	20 (38.5)^***^	95 (15.1)^*^	4 (6.3)	<0.001
	Pain/fever	266 (37.5)	32 (61.5)	210 (33.3)	30 (46.9)	<0.001
	Sedation	35 (4.9)	8 (15.4)^**^	40 (6.4)	1 (1.6)	0.013
Time of onset of illness to presentation in days^∧^		3 (2)	3 (2)	3 (2)	3 (3)	0.645

∧ *Median (interquartile range)*;

# *Mean (standard deviation)*;

* p < 0.05;

** p < 0.01;

*** *p < 0.001 in comparison to group 4 (high-flow room air group)*.

**Table 2:** Change in physiological parameters as a treatment response to oxygen therapy.

**Timepoint**	**Parameter**	**Treatment group**				***p*-value**
		**Standard-oxygen**	**High-flow with immediate oxygen**	**High-flow with oxygen**	**High-flow room air**	
		***N* = 710**	***N* = 52**	***N* = 630**	***N* = 64**	
Pre-enrolment	Heart rate^#^	161.8 (22.1) [700]	165.8 (23.9) [52]	162.8 (21.1) [620]	162.1 (20.6) [63]	0.544
	Respiratory rate^#^	52.7 (12.1) [694]	57.3 (14.0) [52]	53.8 (11.5) [618]	50.4 (10.4) [63]	0.005
	SpO_2_^∧^	89.0 (3.0) [706]	82.0 (5.5) [52]^***^	89.0 (3.0) [624]^**^	90.0 (3.0) [64]	<0.001
Post-enrolment (within 1 h)	Heart rate^#^	163.2 (21.6) [165]	169.5 [30.4] [20]	164.2 [19.4] [149]	153.3 (23.5) [18]	0.120
	Respiratory rate^#^	49.9 (10.5) [161]	58.8 (15.0) [19]	52.2 (10.4) [145]	47.3 (11.5) [18]	
	SpO_2_^∧^	98.0 (4.0) [165]^***^	95.0 (3.0) [20]	95.0 (5.0) [147]	95.0 (2.0) [17]	<0.001
Difference (post-pre)	Heart rate^#^	−4.6 (21.7) [156]	−3.8 (12.2) [20]	−3.0 (19.4) [141]	−10.1 (18.0) [17]	0.573
	Respiratory rate^#^	−3.8 (10.9) [151]	−3.2 (10.0) [19]	−2.2 (11.0) [137]	−0.5 (13.9) [17]	0.504
	SpO_2_^∧^	9.0 (5.0) [161]^***^	14.0 (8.0) [20]^***^	6.0 (6.0) [141]	6.0 (4.0) [17]	<0.001
Length of hospital stay	2.21 (2.05) [710]^***^	3.97 (2.37) [52]^***^	2.55 (2.11) [630]^***^	1.55 (0.98) [64]	<0.001

∧ *Median (interquartile range) [N]*;

# *Mean (standard deviation) [N]*;

* *p < 0.05*;

** *p < 0.01*;

*** *p < 0.001 in comparison to group 4 (high-flow room air group)*.

The length of hospital stay was shortest in infants in the high-flow room air group (Table 2). None of the tested pre-enrolment factors such as age, prematurity, RSV, time of onset of disease to presentation or heart nor respiratory rate were predictive for a success of high-flow room air therapy (Table 3).

**Table 3:** Multivariable analysis.

**Characteristic**		**Bivariable analysis**			**Multivariable analysis**		
		**Odds ratio**	**95% CI**	***p*-value**	**Odds ratio**	**95% CI**	***p*-value**
Age (months)	≤ 3 months	0.66	0.33, 1.32	0.238	0.73	0.36, 1.47	0.372
	>3–6 months	1.51	0.86, 2.64	0.154	1.62	0.92, 2.87	0.095
	>6 months (reference group)	1	–	–	1	–	–
Risk Factors	Prematurity <37 weeks	0.95	0.49, 1.85	0.881	–	–	–
	Respiratory syncytial virus	0.80	0.48, 1.34	0.395	–	–	–
Time of onset of illness to presentation (days) (log-transformed)	0.83	0.57, 1.21	0.341	-	–	–
Pre-enrolment	HR	1.00	0.99, 1.01	0.954	–	–	–
	RR	0.98	0.96, 1.00	0.062	0.98	0.96, 1.00	0.079

None of the infants on high-flow room air required intensive care admission and no adverse events were observed in either the high-flow room air group or those who commenced treatment initially in room air.

For completeness, infants in the standard-oxygen group presenting with pre-enrolment saturations <85% were compared to infants with saturations between 85 and≤92/94% and the comparison is shown in Table 4. Similarly, to the high-flow group, infants in the standard-oxygen arm presenting with pre-enrolment saturations of <85% were younger and had lower body weight. The infants in the standard-oxygen group and presenting with saturations <85% received similarly to the patients in the high-flow with immediate oxygen group, significantly more antibiotics and sedation.

**Table 4:** Comparison of those treated with standard-oxygen <85% and ≥85%.

**Pre-enrolment characteristic**		**Standard oxygen**		***p*-value**
		**SpO_**2**_ <85%**	**SpO_**2**_ ≥85%**	
		***N*** **=** **43**	***N*** **=** **669**	
Age (months)^∧^	4.9 (6.6)	6.4 (5.9)	0.029
Age (months)	≤3 months	18 (43.9)	162 (24.2)	0.024
	>3–6 months	8 (19.5)	152 (22.7)	
	>6 months	15 (36.6)	355 (53.1)	
Weight (kg)^#^	6.7 (2.6)	7.7 (2.2)	0.004
Sex	Female	11 (26.8)	241 (36.0)	0.313
Ethnicity	Caucasian	18 (43.9)	352 (52.6)	0.464
	Aboriginal/Torres Strait Islander	1 (2.4)	28 (4.2)	
	Maori/Pacific Islander	17 (41.5)	196 (29.3)	
	Other/unknown	5 (12.2)	93 (13.9)	
Risk factors	Prematurity <37 weeks	11 (26.8)	112 (16.7)	0.133
	Previous need for neonatal respiratory support	5 (12.2)	96 (14.4)	0.821
	Oxygen only	2 (4.9)	34 (5.1)	0.999
	CPAP/NIV	4 (9.8)	66 (9.9)	0.999
	Invasive ventilation	0 (0.0)	19 (2.8)	0.619
	Other respiratory support	1 (2.4)	11 (1.6)	0.513
	Previous hospital admissions for respiratory disease postnatal	14 (34.2)	203 (30.3)	0.603
	Previous ICU admission for respiratory support	2 (4.9)	44 (6.6)	0.999
	Intubated & ventilated	0 (0.0)	7 (1.1)	0.999
	Mask ventilation	0 (0.0)	6 (0.9)	0.999
	High-flow therapy	2 (4.9)	33 (4.9)	0.999
	Chronic Lung Disease	1 (2.4)	11 (1.7)	0.514
	Congenital Heart Disease	2 (4.9)	14 (2.1)	0.236
	Patient history of wheeze	9 (22.0)	165 (24.8)	0.852
	Family history of asthma	14 (34.2)	335 (50.9)	0.052
	Family history of allergy	10 (25.0)	149 (22.7)	0.701
	Currently attending child care?	2 (5.0)	89 (13.7)	0.148
Viral etiology	Number tested	*N*=32	*N*=532	
	Respiratory syncytial virus	22 (68.8)	287 (54.0)	0.142
	Other viruses	11 (34.4)	183 (34.4)	0.999
	Multiple viruses	9 (28.1)	97 (18.2)	0.166
	No virus detected on nasopharyngeal aspirate	1 (3.1)	107 (20.1)	0.018
Non study treatments received	Any non-study treatments received	29 (70.7)	481 (71.9)	0.859
	Steroids	1 (2.4)	58 (8.7)	0.242
	Nebulized Saline	4 (9.8)	83 (12.4)	0.807
	Bronchodilators	9 (22.0)	202 (30.2)	0.296
	Adrenaline nebulizations	0 (0.0)	2 (0.3)	0.999
	Antibiotics	15 (36.6)	100 (15.0)	0.001
	Pain/fever	18 (43.9)	248 (37.1)	0.408
	Sedation	5 (12.2)	30 (4.5)	0.045
Time of onset of illness to presentation in days^∧^		3 (3)	3 (2)	0.624

∧ Median (interquartile range);

# *Mean (standard deviation)*.

## Discussion

The World Health Organization recommended in 2012 that effective oxygen delivery systems should be a universal standard of care and should be made more widely available (21). At the same time, an increasing body of evidence suggests potential for harm related to oxygen application in various settings including perinatal adaptation, cardiac arrest, and intensive care (2–5). Furthermore, in adults receiving mechanical ventilation it has been shown that a large number of patients were left on unnecessarily high inspired oxygen fraction, which can contribute to lung disease (22). Bronchiolitis represents the most common disease leading to non-elective admission of infants and oxygen remains the mainstay of therapy for this patient group (23). Our data demonstrates that in a small subgroup of hypoxemic infants with bronchiolitis the oxygen requirement can be safely treated with high-flow in room air for their entire hospital stay. Infants responding to high-flow room air not only showed a significant improvement in their saturations but also a reduction in heart and respiratory rates indicating effective respiratory support (Figures 2, 3). The reversal of the hypoxemia was likely due to the positive airway pressure effect generated by nasal high-flow therapy, which improved the underlying pathophysiology such as ventilation maldistribution and loss of lung volume (atelectasis). Previous physiological studies have shown that high-flow rates at 2 L/kg/min generate a positive airway pressure in the range of 4–6 cm H_2_O, which can restore the patient's functional residual capacity and reduce ventilation inhomogeneity (24, 25).

The current recommendations for first line treatment of hypoxemia is the provision of oxygen (26). If supplemental oxygen is ineffective, then as a second line treatment some form of positive pressure ventilation either non-invasive or invasive ventilation should be used. The study design and outcomes in the high-flow arm suggests it is safe to initially institute high-flow in room air and to subsequently supplement oxygen in those who need it, except in infants with severe hypoxemia on presentation (saturation <85%). The results of this prospective trial indicate that a small proportion of infants (10%) will respond to high-flow room air alone, and those requiring oxygen can be safely titrated to the appropriate dose. Albeit a small proportion of infants in our study cohort responded to high-flow room air, these findings provide as a first interesting physiological proof of concept that in these infants oxygenation can be improved by providing positive airway pressure only.

A multivariate analysis did not demonstrate any predictive factors for responders of this approach. Infants who required high-flow room air only had a significantly shorter stay in hospital, most likely as a result of a milder form of the respiratory disease.

The results of our study serve as a proof of concept that may inform future clinical trials particularly in less well-resourced settings where access to oxygen is limited. This could prove beneficial for regions where the mortality of infants with chest infections and an oxygen requirement is 8% (African FEAST trial) (27, 28). A recent study in Bangladesh compared standard-oxygen, bubble CPAP, and high-flow in children with chest infections aged up to 5 years. The median saturation at presentation in this study was 86 % (IQR 82–88) with only 14% of enrolled children having a saturation of <85% (29). The current COAST trial being performed in Uganda and Kenya in children presenting with a chest infection and an oxygen requirement is currently investigating if high-flow in room air can reverse hypoxemia and reduce mortality (28).

One of the shifts in paradigm in this study was that oxygen therapy was started from “low”—in room air—and titrated up to reach the desired SpO_2_ threshold >92/94% whereas traditionally oxygen therapy was introduced from “high” to achieve maximal saturations and then titrated down. It is likely that the titrate up strategy will mean that saturation levels will be maintained closer to the lower acceptable levels than in the titrate down strategy. Hyperoxia is commonly observed in mechanically ventilated patients and most often related to the reluctance of clinicians to reduce the inspired oxygen fraction (22). In the small cohort of infants in whom physiological parameters were also measured within the first hour of treatment, the oxygen saturations were highest in the standard-oxygen group. In the high-flow group the clinicians were instructed to start with low FiO_2_ (room air) and only increase if required, whereas in the standard-oxygen group no instructions were given.

### Limitations

The medical records in the participating sites did not allow measuring the fidelity if clinicians strictly adhered to the proposed high-flow room air approach, hence the proportion of patients who would have benefitted from the high-flow room air approach may have been greater. Our study protocol used a saturation target between 92 and 98% (in some centers 94–98%). WHO is recommending a lower threshold of 90% to initiate oxygen therapy. Therefore, using a lower treatment threshold for hypoxemia would have likely increased the proportion of successful treatments with high-flow in room air. A recent study in bronchiolitis in the UK showed that keeping a threshold of 90% compared to 94% did not impact on safety and efficacy (30).

## Conclusion

A small proportion of infants with bronchiolitis and hypoxemia who were admitted to hospital did respond to nasal high-flow therapy using room air only. The clinical improvement of the high-flow therapy could be demonstrated immediately with decreased heart and respiratory rate. Nasal high-flow room air therapy may be a valuable option to treat hypoxemia in bronchiolitis in settings with limited access to oxygen.

## Evidence Before this Study

A comprehensive literature search using MEDLINE, Cochrane, CINAHL, and EMBASE demonstrated that high-flow therapy has clinical benefit over standard oxygen therapy used in infants with bronchiolitis in general ward settings with two large randomized controlled trials showing a reduced need to escalate therapy. No study could be identified to investigate the role of high-flow in room air and its clinical effect.

## Added Value of this Study

The study findings are a proof of concept. The results may instigate new trials using high-flow in room air in settings with limited access to oxygen supplies.

## Implications of All the Available Evidence

This study presents as a first a novel concept to improve hypoxemia in infants with bronchiolitis. The approach is to first apply positive airway pressure support followed by increasing the oxygen fraction if required. Titration of oxygen from low to normal saturation as a mean to limit possible oxygen toxicity.

## Data Availability Statement

All datasets generated for this study are included in the manuscript/supplementary files.

## Ethics Statement

This study was carried out in accordance with the recommendations of Children's Health Queensland Ethics Committee and with written informed consent from all subjects. All subjects gave written informed consent in accordance with the Declaration of Helsinki. The protocol was approved by Children's Health Queensland Ethics Committee (HREC/13/QRCH/93).

## Author Contributions

All authors listed have made a substantial, direct and intellectual contribution to the work, and approved it for publication.

### Conflict of Interest

The authors declare that the research was conducted in the absence of any commercial or financial relationships that could be construed as a potential conflict of interest.
